# Mechanistic studies of intracellular delivery of proteins by cell-penetrating peptides in cyanobacteria

**DOI:** 10.1186/1471-2180-13-57

**Published:** 2013-03-14

**Authors:** Betty R Liu, Yue-Wern Huang, Han-Jung Lee

**Affiliations:** 1 Department of Natural Resources and Environmental Studies, National Dong Hwa University, Hualien 97401, Taiwan; 2 Department of Biological Sciences, Missouri University of Science and Technology, Rolla, MO 65409-1120, USA

**Keywords:** Cell-penetrating peptide (CPP), Endocytosis, Green fluorescent protein (GFP), Macropinocytosis, Protein transduction, Red fluorescent protein (RFP)

## Abstract

**Background:**

The plasma membrane plays an essential role in selective permeability, compartmentalization, osmotic balance, and cellular uptake. The characteristics and functions of cyanobacterial membranes have been extensively investigated in recent years. Cell-penetrating peptides (CPPs) are special nanocarriers that can overcome the plasma membrane barrier and enter cells directly, either alone or with associated cargoes. However, the cellular entry mechanisms of CPPs in cyanobacteria have not been studied.

**Results:**

In the present study, we determine CPP-mediated transduction efficiency and internalization mechanisms in cyanobacteria using a combination of biological and biophysical methods. We demonstrate that both *Synechocystis* sp. PCC 6803 and *Synechococcus elongatus* PCC 7942 strains of cyanobacteria possess red autofluorescence. Green fluorescent protein (GFP), either alone or noncovalently associated with a CPP comprised of nine arginine residues (R9/GFP complexes), entered cyanobacteria. The ATP-depleting inhibitor of classical endocytosis, *N*-ethylmaleimide (NEM), could block the spontaneous internalization of GFP, but not the transduction of R9/GFP complexes. Three specific inhibitors of macropinocytosis, cytochalasin D (CytD), 5-(*N*-ethyl-*N*-isopropyl)-amiloride (EIPA), and wortmannin, reduced the efficiency of R9/GFP complex transduction, indicating that entry of R9/GFP complexes involves macropinocytosis. Both the 1-(4,5-dimethylthiazol-2-yl)-3,5-diphenylformazan (MTT) and membrane leakage analyses confirmed that R9/GFP complexes were not toxic to the cyanobacteria, nor were the endocytic and macropinocytic inhibitors used in these studies.

**Conclusions:**

In summary, we have demonstrated that cyanobacteria use classical endocytosis and macropinocytosis to internalize exogenous GFP and CPP/GFP proteins, respectively. Moreover, the CPP-mediated delivery system is not toxic to cyanobacteria, and can be used to investigate biological processes at the cellular level in this species. These results suggest that both endocytic and macropinocytic pathways can be used for efficient internalization of regular protein and CPP-mediated protein delivery in cyanobacteria, respectively.

## Background

Cyanobacteria, also known as blue-green algae, are photosynthetic prokaryotes. They played a key role in the evolution of life on Earth, converting the early reducing atmosphere into an oxidizing one as they performed oxygenic photosynthesis [1]. Cyanobacteria are thought to be progenitors of chloroplasts via endosymbiosis [2]. Approximately, 20–30% of Earth's photosynthetic activity is due to cyanobacteria. The proteomic composition and dynamics of plasma membranes of cyanobacteria have been extensively characterized [2,3]. However, the influence of the structure and composition of cyanobacterial membranes on cellular uptake remains largely unknown. Delivery of exogenous DNA into cyanobacteria was first reported in 1970 [4], although the internalization mechanisms are still unknown [1]. Since cyanobacteria play key roles in supporting life on Earth and have potential in biofuel production and other industrial applications [5-7], understanding how they interact with the environment by processes such as internalization of exogenous materials, is becoming increasingly important.

The plasma membrane provides a barrier that hinders the cellular entry of macromolecules, including DNAs, RNAs, and proteins. In 1988, two groups simultaneously identified a protein called transactivator of transcription (Tat) from the human immunodeficiency virus type 1 (HIV-1) that possesses the ability to traverse cellular membranes [8,9]. The penetrating functional domain of the Tat protein is comprised of 11 amino acids (YGRKKRRQRRR) [10]. Subsequently, many peptide analogues of the basic amino acid-rich domain of the Tat protein were synthesized and evaluated for membrane transduction potential [11,12]. These positively charged, amphipathic peptides were termed cell-penetrating peptides (CPPs) or protein transduction domains (PTDs) [11-13].

Among synthetic peptides, the cellular uptake of polyarginine was found to be much more efficient than that of polylysine, polyhistidine, or polyornithine [13,14]. We found that a nona-arginine (R9) CPP peptide can enter cells by itself or in conjunction with an associated cargo [15-21]. Cargoes that R9 can carry include proteins, DNAs, RNAs, and inorganic nanoparticles (notably, quantum dots; QDs). R9 can form complexes with cargoes in covalent, noncovalent, or mixed covalent and noncovalent manners [22-24]. CPPs can deliver cargoes up to 200 nm in diameter [11,25], and R9 can internalize into cells of various species, including mammalian cells/tissues, plant cells, bacteria, protozoa, and arthropod cells [16,17,26,27].

Despite many studies using various biological and biophysical techniques, our understanding of the mechanism of CPP entry remains incomplete and somewhat controversial. Studies have indicated that CPPs enter cells by energy-independent and energy-dependent pathways [28]. The concentration of CPPs appears to influence the mechanism of cellular uptake [28]. Our previous studies indicated that macropinocytosis is the major route for the entry of R9 carrying protein or DNA cargoes associated in a noncovalent fashion [15,29,30]. However, we found that CPP/QD complexes enter cells by multiple pathways [31,32]. Multiple pathways of cellular uptake were also demonstrated with CPP-fusion protein/cargo complexes associated in a mixed covalent and noncovalent manner [22,24]. In contrast, our study of the R9 modified with polyhistidine (HR9) indicated direct membrane translocation [33].

The cellular entry mechanisms of CPPs in cyanobacteria have not been studied. In the present study, we determined CPP-mediated transduction efficiency and internalization mechanisms in cyanobacteria using a combination of biological and biophysical methods.

## Results

### Autofluorescence

**Figure 1:**
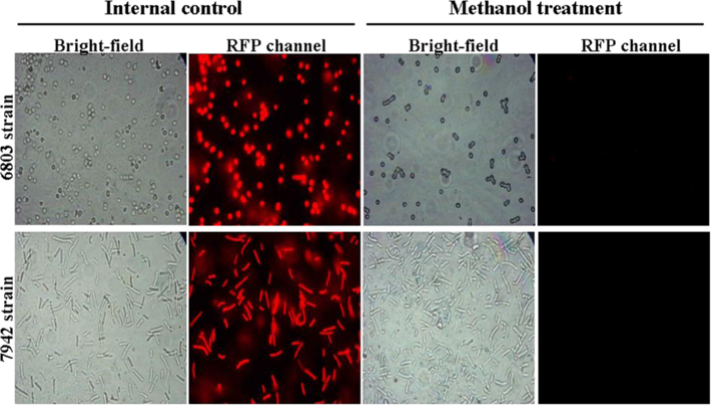
**Autofluorescence detection in 6803 and 7942 strains of cyanobacteria.** Cells were treated with either BG-11 medium or 100% methanol to cause cell death. Bright-field and fluorescent images in the RFP channel were used to determine cell morphology and autofluorescence, respectively. Images were recorded using an Eclipse E600 fluorescent microscope (Nikon) at a magnification of 1,000×.

To detect autofluorescence in cyanobacteria, either live or methanol-killed cells were observed using a fluorescent microscope. Both 6803 and 7942 strains of cyanobacteria emitted red fluorescence under blue or green light stimulation (Figure 1, left panel) when alive; dead cells did not display any fluorescence (Figure 1, right panel). This phenomenon was confirmed using a confocal microscope; dead cyanobacteria treated with either methanol or killed by autoclaving emitted no red fluorescence (data not shown). Thus, red autofluorescence from cyanobacteria provided a unique character.

### Mechanistic studies of protein transduction

**Figure 2:**
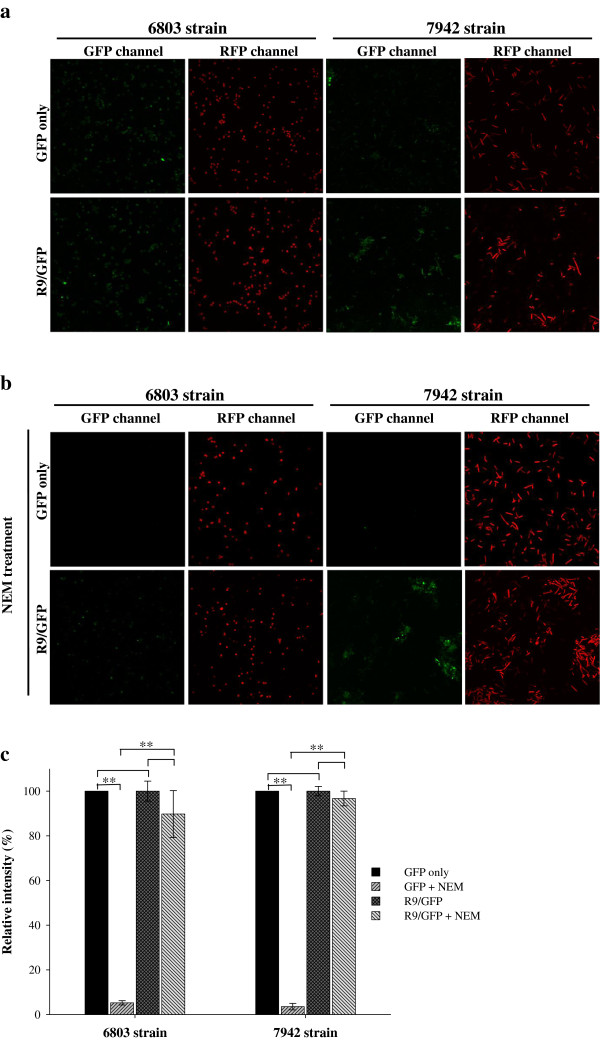
**CPP**-**mediated GFP delivery in cyanobacteria.** (**a**) The 6803 and 7942 strains of cyanobacteria were treated with GFP only or R9/GFP mixtures for 20 min at room temperature. (**b**) GFP delivery in the presence of the endocytic inhibitor NEM. Cells were pretreated with NEM, and then either GFP only or R9/GFP was added to cells for 20 min. Green and red fluorescence were detected in GFP and RFP channels using a Leica confocal microscope at a magnification of 1,000× (**a** and **b**). (**c**) Histogram of relative fluorescent intensity. Green fluorescence detected in the cells treated with only GFP served as a control. Fluorescent intensity detected in experimental groups was compared to that of the control group. Data are presented as mean ± SD from three independent experiments. Significant differences were set at *P* < 0.05 (*) or 0.01 (**).

To demonstrate protein transduction in cyanobacteria, both 6803 and 7942 strains were treated with either green fluorescent protein (GFP) alone or R9/GFP noncovalently complexed at a molecular ratio of 3:1. After 20 min, the medium was removed, and cells were washed and observed using a confocal microscope. Surprisingly, green fluorescence was detected in both control and experimental groups in both strains (Figure 2a). Red autofluorescence indicated that the cells in both groups are alive (Figure 2a). To test whether GFP alone enters cyanobacteria by classical endocytosis, physical and pharmacological inhibitors, including low temperature, valinomycin, nigericin, *N*-ethylmaleimide (NEM), and sodium azide, were used. Endocytic efficiencies of GFP were significantly reduced in the 7942 strain treated with 1 and 2 mM of NEM, while 2 mM of NEM suppressed GFP uptake in the 6803 strain (Additional file 1: Figure S1A). All of these inhibitors reduced the entry of GFP, indicating that endocytosis is the route for spontaneous GFP internalization (Additional file 1: Figure S1B). Insofar as NEM was the most effective inhibitor of classical endocytosis in both stains (Additional file 1: Figure S1B), it was used in subsequent experiments.

To block classical energy-dependent endocytosis in cyanobacteria, NEM was added to cells for 1 min followed by addition of either GFP alone or R9/GFP complexes. We found that both strains treated with GFP emitted red fluorescence but not green fluorescence (Figure 2b). In contrast, both green and red fluorescence were detected in the cells treated with R9/GFP complexes (Figure 2b). Relative fluorescent intensities were analyzed and compared with control cells in the absence of NEM and R9. NEM treatment decreased green fluorescence in cells exposed to GFP alone (Figure 2c), but did not affect the level of green fluorescence in cells treated with R9/GFP mixtures (Figure 2c). These results suggest that GFP cannot cross NEM-treated cell membranes without the assistance of R9. Thus, we hypothesize that there is an alternative route of protein transduction in cyanobacteria in addition to classical endocytosis.

**Figure 3:**
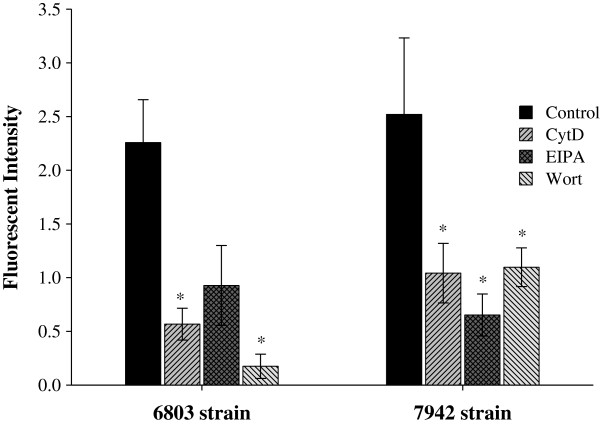
**The mechanism of the CPP**-**mediated GFP delivery in 6803 and 7942 strains of cyanobacteria.** Cells were treated with NEM and R9/GFP mixtures in the absence or presence of CytD, EIPA, or wortmannin (Wort), as indicated. Results were observed in the GFP channel using a confocal microscope, and fluorescent intensities were analyzed by the UN-SCAN-IT software. Data are presented as mean ± SD from three independent experiments. Significant differences of *P* < 0.05 (*) are indicated.

To identify the alternative route for cellular entry of R9/GFP complexes in cyanobacteria, we used macropinocytic inhibitors 5-(*N*-ethyl-*N*-isopropyl)-amiloride (EIPA), wortmannin, and cytochalasin D (CytD) in cells pretreated with NEM to block clathrin- and caveolin-dependent endocytosis. The cells were treated with either R9/GFP as a control or R9/GFP plus macropinocytic inhibitors. Significant reductions in the intensity of cellular green fluorescence were observed in treatments with CytD and wortmannin in the 6803 strain of cells, and with all of the macropinocytic inhibitors in the 7942 strain of cells (Figure 3). Wortmannin was the most effective inhibitor in the 6803 strain, while EIPA was the most effective inhibitor in the 7942 strain (Figure 3). These results indicate that protein transduction of R9 in cyanobacteria involves lipid raft-dependent macropinocytosis.

### Cytotoxicity

**Figure 4:**
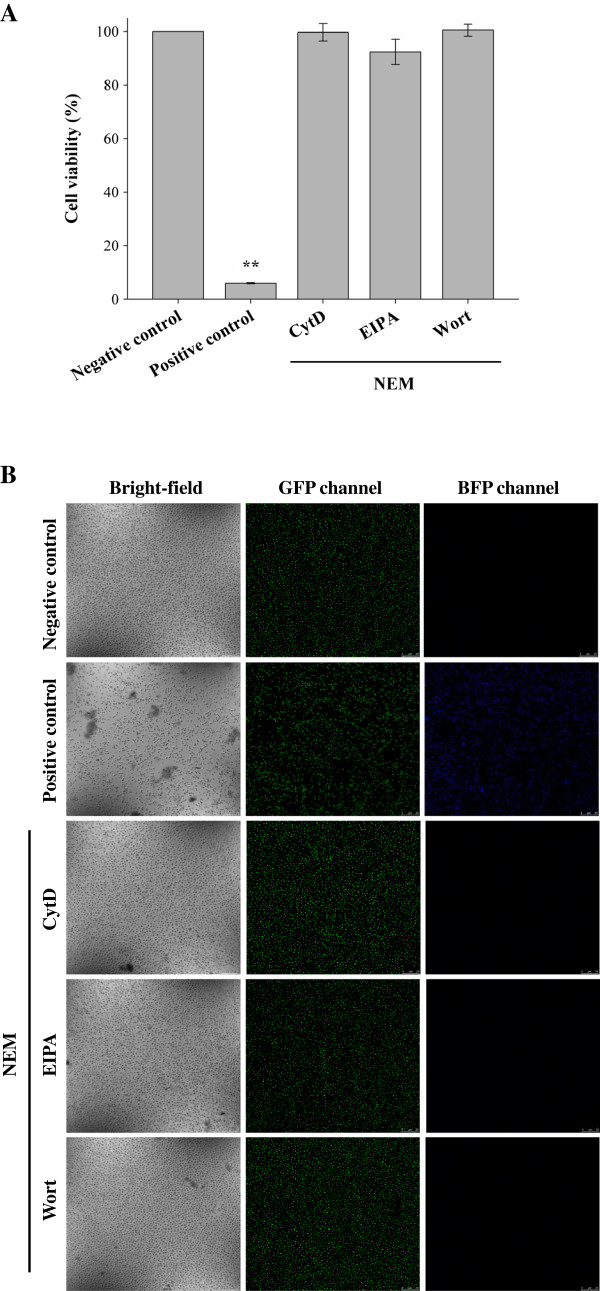
**Cell viability of the R9**/**GFP delivery system in the presence of uptake modulators.** (**A**) The MTT assay. The 6803 strain of cyanobacteria was treated with BG-11 medium as a negative control, or treated with 100% methanol as a positive control. In the presence of NEM, cells were treated with R9/GFP complexes in the presence of CytD, EIPA, or wortmannin (Wort), respectively, and analyzed by the MTT assay. Significant differences were determined at *P* < 0.01 (**). Data are presented as mean ± SD from nine independent experiments. (**B**) The membrane leakage assay by a two-color fluorescence assay. The 6803 strain of cyanobacteria was treated with the same conditions in (**A**). SYTO 9 stains nucleic acids of live and dead cells in the GFP channel, while SYTOX blue stains nucleic acids of membrane-damaged cells in the BFP channel. Blue and green fluorescence were detected in BFP and GFP channels using a Leica confocal microscope at a magnification of 630×.

To investigate whether treatments with R9 and GFP are toxic and cause membrane leakage, cytotoxicity was evaluated using cells treated with BG-11 medium and 100% methanol as negative and positive controls, respectively. In the presence of NEM, cells were incubated with R9/GFP complexes mixed with CytD, EIPA, or wortmannin as experimental groups, respectively. The 1-(4,5-dimethylthiazol-2-yl)-3,5-diphenylformazan (MTT) assay was applied. There is a significant correlation (R^2^ = 0.9949) between cell number and activity of MTT reduction (Additional file 2: Figure S2A). Further, 100% methanol, 100% dimethyl sulfoxide (DMSO), and autoclave treatments were effective in causing cell death (Additional file 2: Figure S2B). We chose 100% methanol treatment as a positive control for cytotoxicity analysis. The 6803 strain treated with R9/GFP complexes mixed with CytD, EIPA, or wortmannin in the presence of NEM was analyzed by the MTT assay. No cytotoxicity was detected in experimental groups, but significant reduction in cell viability was observed in the positive control (Figure 4A). To further confirm the effect of endocytic modulators on cell viability, the membrane leakage assay was conducted. No membrane damage was detected in the negative control and experimental groups (Figure 4B). These data indicate that R9/GFP and endocytic modulators were nontoxic to cyanobacteria.

## Discussion

In this study, we demonstrate that both 6803 and 7942 strains of cyanobacteria use classical endocytosis for protein ingestion. Macropinocytosis is used by R9-mediated delivery system as an alternative route of cellular entry when classical endocytosis is blocked (Figure 2b, 2c, and 3). Our finding of macropinocytosis-mediated entry of a CPP is consistent with studies of protein and DNA delivery in other eukaryotic cells [29,30,34].

We also demonstrate that cyanobacteria possess red autofluorescence. Identification and quantification of cyanobacteria in environmental samples or cultures can be time-consuming (such as plating, fluorescent staining, and imaging) and sometimes costly. Schulze et al. recently presented a new and fast viability assay for the model organism 6803 strain of cyanobacteria [35]. This method used red autofluorescence of 6803 strain of cyanobacteria to differentiate viable cells from nonviable cells without tedious preparation [35-39]. A combination of this new assay with absorption spectra or chlorophyll concentration measurements was further proposed for more accurate quantification of the vitality of cyanobacteria [35].

Most previous reports have focused on photosynthesis as the major route by which cyanobacteria obtain nutrition, while only a handful of studies have evaluated endocytosis as a means of nutrition ingestion [1,40,41]. The first indication of macropinocytosis in cyanobacteria came from our initial screening of CPP-mediated noncovalent protein transduction among some representative organisms [26]. We found that the mechanism of protein transduction in cyanobacteria may involve both classical endocytosis and macropinocytosis [26]. While cyanobacteria contain cell walls and peptidoglycan layers [3], these structures did not hinder the penetration of CPPs in cyanobacteria (Figure 3), Gram-negative bacteria, Gram-positive bacteria and plants [26,42,43]. Our study is the first report that cyanobacteria use both endocytosis and macropinocytosis to internalize exogenous macromolecules (Figures 2 and 3). The sensitivity of cyanobacteria to macropinocytic inhibitors is strain-specific: the 6803 strain is more sensitive to wortmannin, while EIPA is more highly effcetive at reducing protein transduction in the 7942 strain (Figure 3). There was no enough evidence yet to explain why NEM-treated cyanobacteria decreased green fluorescence in cells exposed to GFP alone. We hypothesize that the spontaneous internalization of GFP in cyanobacteria may be mediated heavily by energy-dependent endocytosis, which can be blocked by the ATP depletion reagent NEM (Figures 2 and 3). However, NEM could not completely inhibit CPP-mediated macropinocytosis, which is lipid raft-dependent [25] and may be slightly energy-dependent [44].

Biofuels have emerged as one of promising sources for alternative energy. Initial biofuel development was based on the synthesis of ethanol using fermentative organisms and polysaccharides [1]. The limited availability of polysaccharides led to extensive research on the direct use of sunlight, the ultimate energy source on this planet. Photosynthetic microorganisms can accomplish this by fixing carbon dioxide and converting sunlight energy into chemical energy as fuel. This raises the possibility of using engineered cyanobacteria in two ways to improve phtotosynthetic biofuel production. Cyanobacteria could be either gene-engineered using recombinant DNA technology [45,46] or protein-engineered using CPP-mediated protein delivery method. Cyanobacteria have an advantage compared to eukaryotic algae in that the genetic manipulation of cyanobacteria is more straightforward and well-developed [1,45]. However, the protein engineering of cyanobacteria mediated by CPPs is just at its infancy.

## Conclusions

In this study, we have demonstrated that both *Synechocystis* sp. PCC 6803 and *Synechococcus elongatus* PCC 7942 strains of cyanobacteria possess red autofluorescence. Cyanobacteria use classical endocytosis and macropinocytosis to internalize exogenous GFP and CPP/GFP proteins, respectively. Moreover, the CPP-mediated delivery system is not toxic to cyanobacteria, and can be used to investigate biological processes at the cellular level in this species.

## Methods

### Culture of cyanobacteria

*Synechocystis* sp. PCC 6803 (American Type Culture Collection, Manassas, VA, USA, 27184) and *Synechococcus elongatus* PCC 7942 (ATCC, 33912) were grown in BG-11 medium with mild shaking at 50 rpm and regular illumination at 28°C, as previously described [26].

### Plasmid construction and protein preparation

We used a pR9 plasmid containing a hexa-histidine and an R9 sequence under the control of the T7 promoter, as previously described [42]. The pQE8-GFP plasmid consisted of the coding sequence of *GFP* under the control of the T5 promoter [42]. Plasmid DNA was purified using a Nucleobond AX100 Kit (Machery-Nagel, Duren, Germany).

Both pR9 and pQE8-GFP plasmids were transformed into *Escherichia coli* and induced, as previously described [47]. The expressed proteins were purified by one-step immobilized-metal chelating chromatography. The purified proteins were concentrated and dialyzed using the Amicon Ultra-4 centrifugal filter devices (Millipore, Billerica, MA, USA), as previously described [15]. Proteins were then quantified using a Protein Assay Kit (Bio-Rad, Hercules, CA, USA).

### Protein transduction and mechanism of cellular uptake

The purified R9 peptide was mixed with GFP at a molecular ratio of 3:1 at room temperature for 10 min. To investigate the delivery of exogenous proteins into cyanobacteria, cells were washed with double deionized water and treated with either GFP alone at a final concentration of 800 nM or R9/GFP mixtures at a molecular ratio of 3:1. To determine the transduction of noncovalent protein complexes, 1 and 2 mM of NEM (Sigma-Aldrich, St. Louis, MO, USA) was added to cyanobacteria, and either GFP alone or R9/GFP mixtures were then added to cyanobacteria for 20 min [26].

To evaluate the role of classical endocytosis, physical and pharmacological inhibitors, such as low temperature, 2 μM of valinomycin [48], 2 μM of nigericin [49], 1 and 2 mM of NEM [50], 10 μM of fusicoccin [51], and 10 mM of sodium azide [49], were used, as previous described [31-33,52]. To study macropinocytosis, cells were treated with or without 100 μM of EIPA (Sigma-Aldrich), 10 μM of CytD (Sigma-Aldrich), or 100 nM of wortmannin (Sigma-Aldrich) followed by the treatment of R9/GFP mixtures [31-33,52]. CytD is a blocker of the F-actin rearrangement that disrupts all forms of endocytosis, including clathrin-, caveolae-dependent endocytosis, and macropinocytosis [31,33]. EIPA is an inhibitor of the Na^+^/H^+^ exchanger and specifically inhibits macropinocytosis [31,53]. Wortmannin interrupts the action of phosphoinositide 3-kinase, which plays the key role in macropinocytosis [53,54]. Protein transduction was quantified by fluorescent and confocal microscopy.

### Cytotoxicity assay

Cyanobacteria were treated with either BG-11 medium or 100% methanol [55] for 24 h as a negative or positive control, respectively. The MTT assay was used to determine cell viability [16,56]. Cells were treated with 100% methanol, 100% DMSO, autoclave, or R9/GFP complexes in the presence of endocytic modulators, and then the MTT assay was performed. For the membrane leakage assay, cyanobacteria were treated with BG-11 medium as a negative control, treated with 100% methanol as a positive control, or R9/GFP complexes in the presence of endocytic modulators. After a 24 h incubation, cells were washed with double-deionized water three times and then stained with 5 μM of either SYTO 9 (LIVE/DEAD BacLight Bacterial Viability Kit, Molecular Probes, Eugene, OR, USA) or SYTOX blue (Invitrogen, Carlsbad, CA) [57] for 30 min at room temperature. SYTO 9 stains nucleic acids of live and dead prokaryotes in green fluorescence. SYTOX blue does not cross the membranes of live cells, whereas the nucleic acids of membrane-damaged cells fluoresce bright blue by SYTOX blue. After washing with double-deionized water, cells were observed using the TCS SP5 II confocal microscope system (Leica, Wetzlar, Germany).

### Fluorescent and confocal microscopy and autofluorescence observation

Both bright-field and fluorescent images were observed using an Eclipse E600 fluorescent microscope (Nikon, Melville, NY, USA) and recorded using a Penguin 150CL cooled CCD camera (Pixera, Los Gatos, CA, USA), as previously described [58]. Confocal fluorescent images were obtained using both the TCS SL as previously described [24,59] and SP5 II confocal microscope systems (Leica). The parameters of the TCS SL confocal microscopy were set as follows: excitation at 488 nm and emission at 500–530 nm for the detection of GFP, and excitation at 543 nm and emission at 580–650 nm for the detection of red fluorescent protein (RFP). Intensities of fluorescent images were quantified using UN-SCAN-IT software (Silk Scientific, Orem, UT, USA). The parameters of the TCS SP5 II confocal microscopy were set as follows: excitation at 405 nm and emission at 436–480 nm for the detection of blue fluorescent protein (BFP), and excitation at 488 nm and emission at 498–523 nm for the detection of GFP.

For autofluorescence observation, cyanobacteria were treated with either BG-11 medium or 100% methanol for 24 h. The cells were then washed with double deionized water three times followed by microscopic observation.

### Statistical analysis

Results are expressed as mean ± standard deviation (SD). Mean values and SDs were calculated from at least three independent experiments carried out in triplicates in each group. Statistical comparisons between the control and treated groups were performed by the Student's *t*-test, using levels of statistical significance of *P* < 0.05 (*) and *P* < 0.01 (**), as indicated.

## Abbreviations

BFP: Blue fluorescent protein; CPP: Cell-penetrating peptide; CytD: Cytochalasin D; DMSO: Dimethyl sulfoxide; EIPA: 5-(*N*-ethyl-*N*-isopropyl)-amiloride; GFP: green fluorescent protein; MTT: 1-(4,5-dimethylthiazol-2-yl)-3,5-diphenylformazan; NEM: *N*-ethylmaleimide; QD: Quantum dot; R9: Nona-arginine; RFP: Red fluorescent protein; Tat: Transactivator of transcription; SD: Standard deviation.

## Competing interests

All authors declare no competing interests.

## Authors’ contributions

BRL performed all experiments and drafted the manuscript. YWH participated in the study design and helped drafting the manuscript. HJL conceived the study idea and assisted in drafting the manuscript. All authors read, commented, and approved the manuscript.

## Supplementary Material

Additional file 1: Figure S1 Endocytic inhibition in cyanobacteria. (A) Endocytic efficiency in cyanobacteria treated with NEM. Both 6803 and 7942 strains were treated with either 1 mM or 2 mM of NEM, followed by the treatment of GFP. (B) Endocytic efficiency in cyanobacteria treated with various endocytic modulators. Low temperature, 2 mM of NEM, 10 μM of fusicoccin, 2 μM of valinomycin, 2 μM of nigericin, and 10 mM of sodium azide were used as the physical and pharmacological inhibitors. Cells were treated with these inhibitors, followed by the treatment of GFP. Significant differences were set at *P* < 0.05 (*) and *P* < 0.01 (**). Data are presented as mean ± SD from three independent experiments. Click here for file

Additional file 2: Figure S2 Cell viability analysis by the MTT assay. (A) Cell number determined by *optical density* (*OD*) at the wavelength of 600 nm linearly correlates with that assessed by the MTT assay at the wavelength of 570 nm. (B) Physical or chemical treatments reduce cell viability. The 6803 strain of cyanobacteria was treated with 100% methanol, 100% DMSO, or autoclave, followed by the MTT assay. Physical or chemical treatment groups were compared with the group without any treatment. And chemical treatment groups were compared with the autoclave group. Significant differences were determined at *P* < 0.01 (**). Data are presented as mean ± SD from nine independent experiments. Click here for file
