# Supplementary material for: Mechanistic studies of intracellular delivery of proteins by cell-penetrating peptides in cyanobacteria
Source: BMC Microbiol. 2013 Mar 14;13:57. doi: 10.1186/1471-2180-13-57 (PMC3637573; doi:10.1186/1471-2180-13-57)
Supplement: Additional file 1: Figure S1 — Endocytic inhibition in cyanobacteria. (A) Endocytic efficiency in cyanobacteria treated with NEM. Both 6803 and 7942 strains were treated with either 1 mM or 2 mM of NEM, followed by the treatment of GFP. (B) Endocytic efficiency in cyanobacteria treated with various endocytic modulators. Low temperature, 2 mM of NEM, 10 μM of fusicoccin, 2 μM of valinomycin, 2 μM of nigericin, and 10 mM of sodium azide were used as the physical and pharmacological inhibitors. Cells were treated with these inhibitors, followed by the treatment of GFP. Significant differences were set at P < 0.05 (*) and P < 0.01 (**). Data are presented as mean ± SD from three independent experiments. [file 1471-2180-13-57-S1.docx]

**Supplementary data**

**Figure S1**


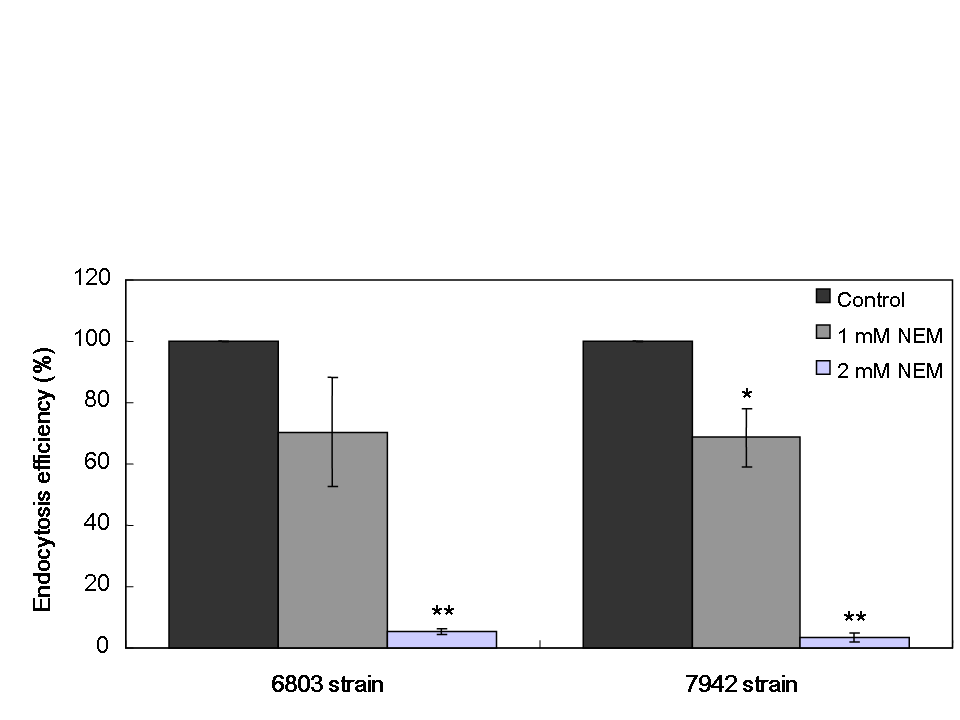


**A**

**B**


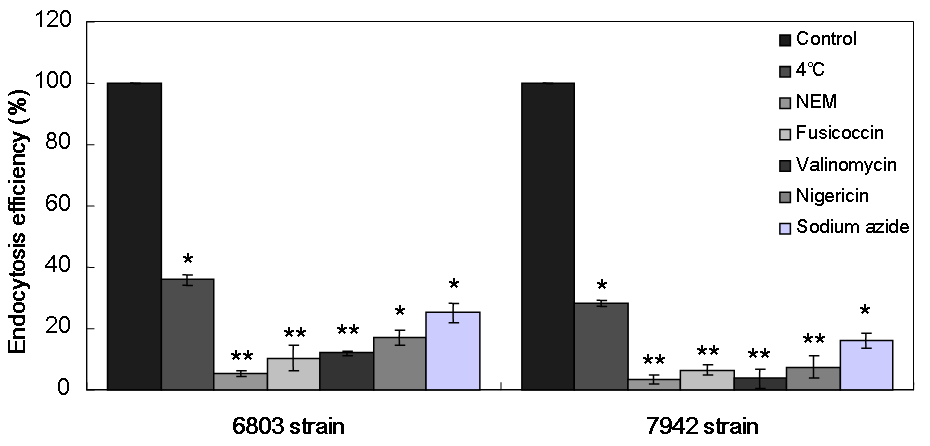


**Additional file 1: Figure S1** **Endocytic inhibition in cyanobacteria.** **(A)** Endocytic efficiency in cyanobacteria treated with NEM. Both 6803 and 7942 strains were treated with either 1 mM or 2 mM of NEM, followed by the treatment of GFP. **(B)** Endocytic efficiency in cyanobacteria treated with various endocytic modulators. Low temperature, 2 mM of NEM, 10 μM of fusicoccin, 2 μM of valinomycin, 2 μM of nigericin, and 10 mM of sodium azide were used as the physical and pharmacological inhibitors. Cells were treated with these inhibitors, followed by the treatment of GFP. Significant differences were set at *P* < 0.05 (*) and *P* < 0.01 (**). Data are presented as mean ± SD from three independent experiments.
