# Supplementary material for: Mechanistic studies of intracellular delivery of proteins by cell-penetrating peptides in cyanobacteria
Source: BMC Microbiol. 2013 Mar 14;13:57. doi: 10.1186/1471-2180-13-57 (PMC3637573; doi:10.1186/1471-2180-13-57)
Supplement: Additional file 2: Figure S2 — Cell viability analysis by the MTT assay. (A) Cell number determined by optical density (OD) at the wavelength of 600 nm linearly correlates with that assessed by the MTT assay at the wavelength of 570 nm. (B) Physical or chemical treatments reduce cell viability. The 6803 strain of cyanobacteria was treated with 100% methanol, 100% DMSO, or autoclave, followed by the MTT assay. Physical or chemical treatment groups were compared with the group without any treatment. And chemical treatment groups were compared with the autoclave group. Significant differences were determined at P < 0.01 (**). Data are presented as mean ± SD from nine independent experiments. [file 1471-2180-13-57-S2.docx]

**Figure S2**


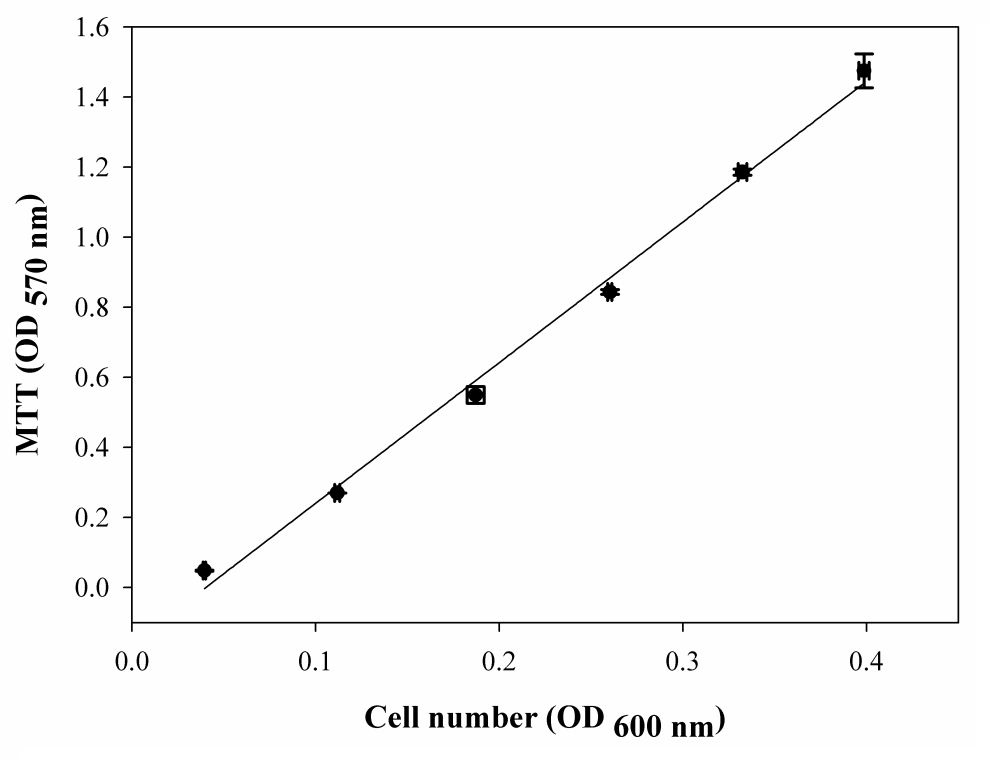


**A**

**B**


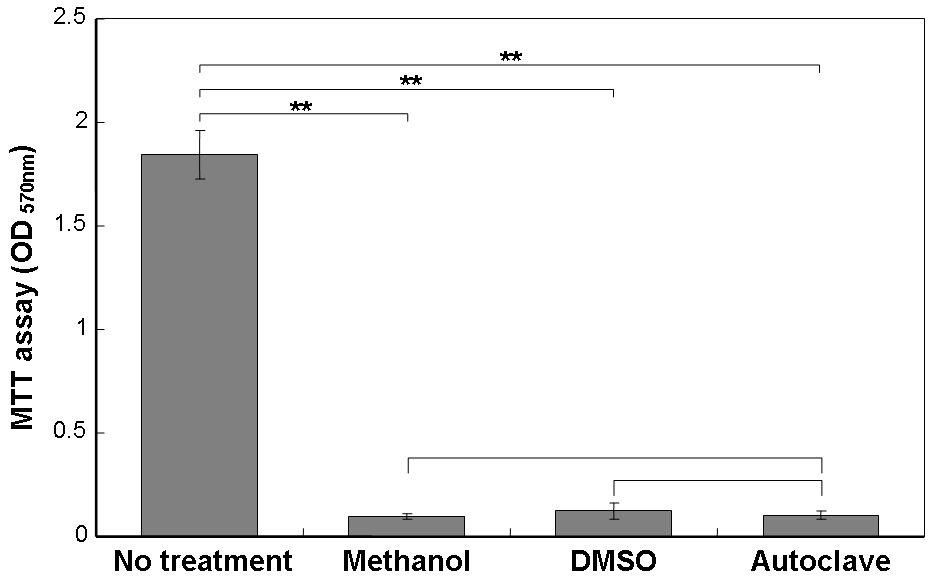


**Additional file 2: Figure S2** **Cell viability analysis by the MTT assay. (A)** Cell number determined by optical density (OD) at the wavelength of 600 nm linearly correlates with that assessed by the MTT assay at the wavelength of 570 nm. **(B)** Physical or chemical treatments reduce cell viability. The 6803 strain of cyanobacteria was treated with 100% methanol, 100% DMSO, or autoclave, followed by the MTT assay. Physical or chemical treatment groups were compared with the group without any treatment. And chemical treatment groups were compared with the autoclave group. Significant differences were determined at *P* < 0.01 (**). Data are presented as mean ± SD from nine independent experiments.
